# Association between Body Mass Index and frequency and grade of varicocele in southeast Iran

**Published:** 2015

**Authors:** F Fazeli, M Shahraki, MM Bazzaz, K Fazeli

**Affiliations:** * Department of Urology, Ali-ebn-Abitaleb Hospital, Zahedan University of Medical Sciences, Zahedan, Iran; ** Department of Nutrition, Faculty of Medicine; Research Center for Children and Adolescent Health, Zahedan University of Medical Sciences, Zahedan, Iran; *** School of Medicine, Mashhad University of Medical Sciences, Mashhad, Iran; **** Department of Community Medicine, School of Medicine, Mashhad University of Medical Sciences, Mashhad, Iran

**Keywords:** Varicocele, Iran, body mass index, grade, Iran

## Abstract

**Background:** Varicocele is associated with impaired spermatogenesis. It may also be associated with the patients’ weight. We aimed to determine the relationship between body mass index (BMI) and the frequency and grade of varicocele among a sample of Iranian men in southeast Iran.

**Methods:** This cross-sectional study was conducted during 2010 and 2011 among 167 men who referred to the University affiliated Urology Clinics in Zahedan, Iran. Expert urologists examined the patients for the existence of varicocele and determined its grade as I to III. The age, grade of varicocele, and the side of varicocele as well as the weight and height of the patients were measured and recorded. The patients were categorized in different age groups. Data were analyzed by using SPSS software.

**Results:** The patients’ mean age was 27.9 ± 7.6 years and their mean BMI was 23.5 ± 4.7 kg/ m2. Varicocele was more frequent in the age group of 20-30 years. Most patients had grade II varicocele without a significant difference between the age groups (P=0.11). The mean BMI of patients with right varicocele was not different from those with left varicocele (P=0.34). The BMI of patients with bilateral varicocele was not different with those having right (P=0.94) and left (P=0.17) varicocele. 17.9%, 56.5%, and 25.6% of the patients had varicocele grade I, II, and III, respectively.

**Conclusions:** In patients with lower BMI, the testicular vein may have a higher grade of varicocele. Urologists should consider examining young, tall men to detect various grades of varicocele as a part of their physical examination.

## Introduction

Varicocele is the dilatation and tortuosity of the pampiniform plexus, which is the network of veins that drain the testicle. It is an abnormal enlargement of the veins located in the scrotum. When the valves within the veins along the spermatic cord do not work appropriately, idiopathic varicocele occurs. The backflow of blood into the pampiniform plexus increases the pressure, and may damage the testicular tissue. In 25-50% of the cases, varicocele is idiopathic. It develops slowly and may be asymptomatic. It is most frequently diagnosed when a patient is 15-30 years old, and occasionally develops after the age of 40. About 98% of the idiopathic varicoceles occur on the left side. This fact might be because the left testicular vein connects to the renal vein while the right testicular vein drains at less than 90-degrees directly into the significantly larger inferior vena cava [**1**,**2**].

Varicocele is the most common cause of surgically treatable infertility in men. It occurs in 15% of men, and in 40% of infertile men. Bilateral varicocele is seen in less than 10% of healthy people, but it is seen in 20% of infertile individuals. Testicular atrophy and impaired semen quality are the main complications of the varicocele [**1**]

Some studies have suggested an influence of the body size on varicocele. In one study, varicocele was reported as the most common abnormal clinical finding in non-fertile, tall, and slim men [**3**]. Some other studies reported an inverse relationship between the prevalence of varicocele and the body mass index (BMI) [**4**,**5**]. Another study demonstrated a higher prevalence of varicocele among adolescents with higher weight and height, but lower BMI than in their counterparts [**6**]. A statistically inverse association between the indexes of generalized and abdominal obesity and the prevalence and severity of varicocele has also been reported. The mentioned study showed that obesity might result in a decreased nutcracker effect, and in turn prevented renal vein compression by the adipose tissue [**7**].

We aimed to determine the association between the BMI and the frequency and grade of varicocele in a sample of Iranian men in southeast Iran.

## Methods

This cross-sectional study was conducted during 2010 and 2011 in Zahedan city, southeast Iran. It comprised the men with varicocele who referred to the Urology Clinics of two teaching hospitals (Imam Ali and Khaatam) affiliated to Zahedan University of Medical Sciences.

All patients with varicocele were recruited during the mentioned period by using the convenient sampling method. The exclusion criteria consisted of conditions with direct and acute effect on BMI such as type 2 diabetes mellitus, type 1 diabetes mellitus under treatment with sulfonylurea, patients receiving systemic corticosteroids, and patients with sudden weight loss such as those with tuberculosis or malignancies (documented by sputum smear, sonography, and other necessary paraclinical studies).

The age, grade of varicocele, and the side of varicocele were recorded in a checklist. Weight and height were measured barefoot and with light clothing by using a calibrated scale and stadiometer (Seca, Japan). BMI was calculated as weight (kg) divided by height squared (m2). The patients were categorized in different age groups.

Overall, 167 patients suspected of having varicoceles were examined by urologists. The following grades were considered for patients: Grade I (small) was considered when varicocele was invisible and only palpable with Valsalva maneuver, grade II (medium) when it was invisible but palpable without Valsalva maneuver, and grade III (large) when it was visible [**1**,**2**].

***Statistical analysis***

Data were analyzed by using SPSS software (SPSS Inc., Chicago, IL, USA). The quantitative data were presented as mean and standard deviation. The independent t and analysis of variance (ANOVA) tests were used for comparison. A P value <0.05 was considered as statistically significant.

## Results

The patients had a mean age of 27.9 ± 7.6 years, (range: 17 to 55 years). Mean BMI of the patients was 23.5 ± 4.7 kg/ m2, (range: 13.6 to 39.8 kg/ m2).

As presented in **Table 1**, the highest frequency of varicocele was seen in the age group of 20-30 years and most patients had grade II without any significant difference between the different age groups (P = 0.11).

**Table 1:** Frequency of different grades of varicocele in different age groups

Grade	I	II	III	Total	
Age (years)	Number (%)	Number (%)	Number (%)	Number (%)	P value
Below 20	3 (16.7)	8 (44.4)	7 (38.9)	18 (100)	0.11
20-30	17 (16.8)	53 (52.5)	31 (30.7)	101 (100)	
30-40	8 (20.0)	29 (72.5)	3 (7.5)	40 (100)	
Over 40	2 (25.0)	4 (50.0)	2 (25.0)	8 (100)	
Total	30 (18.0)	94 (56.3)	43 (25.7)	167 (100)	

The mean ± SD BMI of patients with right varicocele was not significantly different from those with left varicocele (23.2 ± 3.0 vs. 24.6 ± 4.2 kg/ m2, respectively, P=0.34). Likewise the mean ± SD BMI of patients with bilateral varicocele (23.1 ± 4.8 kg/ m2) was not significantly different from those having right (P=0.94) and left (P=0.17) varicocele.

**Fig. 1:**
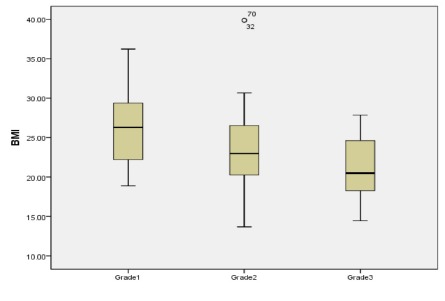
Mean and interquartile range of body mass index according to the grade of varicocele

30 (17.9%) patients had grade I varicocele. The mean ± SD BMI in this group was 26.3 ± 1.4 kg/ m2. 94 (56.3%) patients had grade II varicocele, with a mean ± SD BMI of 23.5 ± 4.7 kg/ m2. 43 (25.6%) patients had grade III varicocele, their mean BMI was 21.2 ± 3.6 kg/ m2 (**Table 2**). **Fig. 1** shows the mean and interquartile BMI range according to the grade of varicocele.

**Table 2:** The comparison between mean body mass Index in patients with various grades of varicocele

	Body mass index		
Varicocele grade	Mean	SD	P value
I	26.3	4.1	0.004
II	23.5	4.7	
Varicocele grade	Mean	SD	P value
I	26.3	4.1	< 0.0001
III	21.7	3.6	
Varicocele grade	Mean	SD	P value
II	23.5	4.7	0.006
III	21.7	3.6	

## Discussion

We found an inverse association between BMI and the grade of varicocele. The mean BMI of our patients (23.5kg/ m2) was higher than that documented in Chen and Haung’s study (22.8kg/ m2) [**7**], and lower than that in Gokce and colleagues’ study (25.1kg/ m2)[**3**].

Some studies showed a higher prevalence of varicocele among individuals with a low BMI [**3**],[**5**]. In a study conducted in 2009 in Taiwan, the biochemical factors and BMI of 102 patients with varicocele were compared with 95 controls. Patients with varicocele had lower cholesterol levels than the healthy controls. The prevalence of varicocele in men with a low BMI was significantly higher than that of the others [**7**]. Consistent with these findings, Tsao and colleagues also showed that the prevalence and severity of varicocele were inversely associated with generalized and abdominal obesity [**8**]. The findings of our study are in line with the aforementioned studies. We found that the mean BMI was significantly higher in patients with grade I than in those with grades II and III of varicocele, and the mean BMI was inversely associated with the severity of varicocele.

Because of anatomical reasons, the most common location for the occurrence of varicocele is in the left testicle. It was documented that more than 80% of the men with left varicocele are prone to bilateral varicocele. In our study, most patients (78.7%) had bilateral varicocele, 14.2% had left side varicocele, and 7.1% had right side varicocele. The mean BMI was not significantly different in patients with right or left varicocele. These findings are consistent with some previous studies [**9**],[**10**].

The inverse association of BMI and varicocele is well documented [**11**-**13**]. Some researchers suggested that the lower prevalence of varicocele in obese persons might be because of increased adipose tissue, making physical examination difficult. However, a study conducted among 1079 patients, of whom 330 (30.6%) had varicocele detected by ultrasound, did not confirm this hypothesis [**14**].

A study conducted among a population-based sample of 1050 young men revealed an inverse relationship between obesity and varicocele. This study confirmed that obesity might result in a decreased nutcracker effect, which may prevent renal vein compression by the adipose tissue [**15**].

In our present study, the highest frequency of varicocele was documented in the age group of 20-30 years. The mean age of patients who had varicocele in a previous study in Iran was higher [**16**],[**17**].

## Conclusion

In patients with lower BMI, the testicular veins may have a higher grade of varicocele. In other words, men with a higher grade of varicocele, have a lower BMI. Urologists should consider examining young, tall men to detect various grades of varicocele as a part of their physical examination
